# The Eleventh Annual Session of the Washington State Dental Society

**Published:** 1898-06

**Authors:** D. I. Burkhart


### NOTICES.

### The Eleventh Annual Session of the Washington State Dental Society

Convened at Tacoma, May 16th to 18th, inclusive, and was held in the rooms of the Tocoma College of Dental Surgery, with President Dr. R. B. Gentle of Seattle, in the Chair. A very interesting meeting was held and those in attendance returned to their work better qualified for their profession and bound with stronger ties socially to their fellows. Oregon and British Columbia were represented on the program.

The officers of the ensueing year are as follows: Dr. P. H. Carlyon, of Olympia, President; Dr. W. E. Burkhart, of Tocoma, 1st Vice-President; Dr. J. N. Prather, of Seattle, 2nd Vice-President; Dr. D. I. Burkhart, of Seattle, Secretary; Dr. J. E. Banks, of North Yakima, Treasurer.

Yours truly,

D. I. BURKHART, *Sec'y.*

